# Correlation of MADRS and YMRS in patients with Bipolar II depression: A post-hoc analysis of an Indian Phase 3 study

**DOI:** 10.1192/j.eurpsy.2025.1072

**Published:** 2025-08-26

**Authors:** A. Dharmadhikari, P. K. Chaurasia, Y. Patel, D. Choudhary, P. L. Dasud, M. Bhirud, P. S. Meena, F. Shah, G. Ganesan, B. P. S. Rathour, K. Mistry, M. Dutta, A. Ramaraju, S. B. Mangalwedhe, S. G. Goyal, G. Kulkarni, A. Mukhopadhyay, P. Chaudhary, G. T. Harsha, M. Parikh, S. Dey, S. Sarkhel, N. U. Jyothi, A. Kumar, N. K. Sooch, A. Shetty, S. Saha, P. H. Devkare, A. Shetty, D. Patil, P. Ghadge, A. Mane, S. Mehta

**Affiliations:** 1 Shree Ashirwad Hospital, Dombivali; 2 Gangoshri Hospital, Varanasi; 3 VS General Hospital, Ahmedabad; 4 GSVM Medical College, Kanpur; 5 Global 5 Hospital, Vashi; 6 Dhadiwal Hospital, Nashik; 7 Jawahar Lal Nehru Medical College, Ajmer; 8 Health 1 Super Speciality Hospital, Ahmedabad; 9 Medstar Speciality Hospital, Bangalore; 10 Atmaram Child Care and Critical Care Hospital, Kanpur; 11 Prajna Health Care, Ahmedabad; 12 Om Hospital, Raipur; 13 Harshamitra Super Speciality Cancer Center and research institute, Trichy; 14 Karnataka Institute of Medical Sciences, Hubli; 15 S. P. Medical College & A.G. Of Hospitals, Bikaner; 16 Manodnya Nursing Home, Sangli; 17 Nil Ratan Sircar Medical College and Hospital, Kolkata; 18 GMERS Medical College, Ahmedabad; 19 Rajlaxmi Hospital, Bangalore; 20 B.J. Medical College and Civil Hospital, Ahmedabad; 21 Sparsh Hospital, Bhubaneswar; 22 IPGME&R and SSKM Hospital, Kolkata; 23 Government General Hospital, Guntur; 24 S N Medical College, Agra; 25 Dayanand Medical College & Hospital, Ludhiana; 26 Sun Pharma, Mumbai, India

## Abstract

**Introduction:**

Lumateperone, an atypical antipsychotic drug approved in 2021 for bipolar depression, has a dual mechanism of action by combination of activity at central serotonin (5-HT2A) and dopamine (D2) receptors. In India, Quetiapine is one of the approved drugs for use in depressive episodes for bipolar disorder.

**Objectives:**

This post-hoc analysis of an Indian Phase 3 study was conducted to evaluate the correlation of severity of depression assessed via Montgomery-Asberg depression rating scale (MADRS) and severity of hypomania symptoms via Young Mania Rating Scale (YMRS) when treated with Lumateperone 42mg or Quetiapine 300mg.

**Methods:**

The phase-III, randomized, multi-centric, assessor-blind, parallel-group, active-controlled, comparative, non-inferiority study included patients with Bipolar II depression with moderate severity having a MADRS score ≥20 and Clinical global impression–bipolar version–severity (CGI-BP-S) score ≥4. The study was conducted after receiving regulatory and ethics committee approvals. The patients were randomized (1:1) to either receive Lumateperone 42mg [Test] or Quetiapine 300mg [Comparator] for 6 weeks. In this post-hoc analysis, correlation between MADRS and YMRS were evaluated and for safety outcomes treatment emergent adverse events (TEAEs) were assessed. [Clinical trial registration: CTRI/2023/10/058583]

**Results:**

This post-hoc analysis included 462 patients [231 each in Test and Comparator]. The baseline demographic characteristics were comparable in between treatment arms. The Pearson’s correlation coefficient between change from baseline in MADRS score and YMRS score was statistically significant for both treatment arms at Day 42 [Test: 0.3144, p<0.0001; Comparator: 0.3284, p<0.0001] and the linear regression between 2 arms was not statistically significant (p=0.4203), indicating weak positive correlation between the 2 scales [Figure 1 and Figure 2]. The incidence of TEAEs were similar in both treatment arms [Test: 34.6%; Comparator: 35.5%] and no serious adverse events were reported.

**Image 1:**

**Figure:**
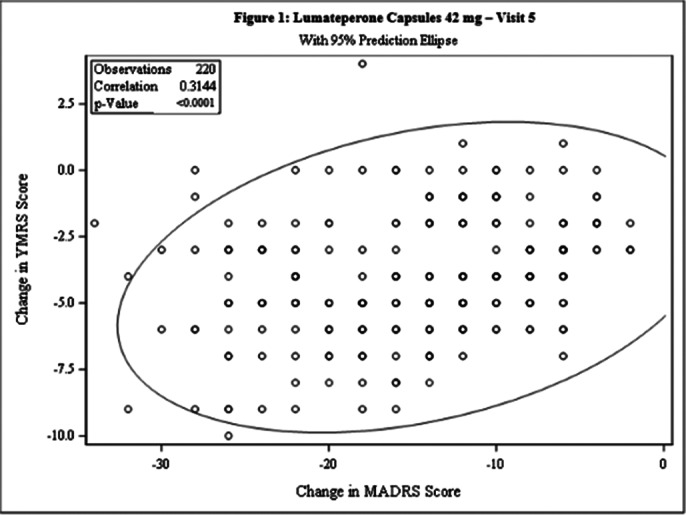

**Image 2:**

**Figure:**
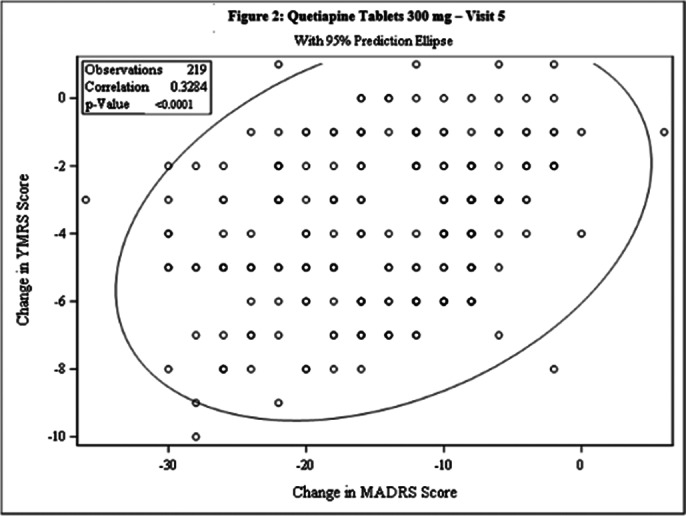

**Conclusions:**

This post-hoc analysis demonstrated that patients with Bipolar II depression when treated with Lumateperone 42mg or Quetiapine 300mg, the reduction in MADRS score is directly proportional to reduction in YMRS score and both treatments were well tolerated.

**Disclosure of Interest:**

A. Dharmadhikari: None Declared, P. Chaurasia: None Declared, Y. Patel: None Declared, D. Choudhary: None Declared, P. Dasud: None Declared, M. Bhirud: None Declared, P. Meena: None Declared, F. Shah: None Declared, G. Ganesan: None Declared, B. P. Rathour: None Declared, K. Mistry: None Declared, M. Dutta: None Declared, A. Ramaraju: None Declared, S. Mangalwedhe: None Declared, S. G. Goyal: None Declared, G. Kulkarni: None Declared, A. Mukhopadhyay: None Declared, P. Chaudhary: None Declared, G. T. Harsha: None Declared, M. Parikh: None Declared, S. Dey: None Declared, S. Sarkhel: None Declared, N. Jyothi: None Declared, A. Kumar: None Declared, N. Sooch: None Declared, A. Shetty Employee of: Sun Pharma, S. Saha Employee of: Sun Pharma, P. Devkare Employee of: Sun Pharma, A. Shetty Employee of: Sun Pharma, D. Patil Employee of: Sun Pharma, P. Ghadge Employee of: Sun Pharma, A. Mane Employee of: Sun Pharma, S. Mehta Employee of: Sun Pharma

